# Supplementary material for: Enhancing Trauma Care: Machine Learning-Based Photoplethysmography Analysis for Estimating Blood Volume During Hemorrhage and Resuscitation
Source: Bioengineering (Basel). 2025 Jul 31;12(8):833. doi: 10.3390/bioengineering12080833 (PMC12383621; doi:10.3390/bioengineering12080833)
Supplement: Supplementary file 1 [file bioengineering-12-00833-s001.zip › bioengineering-3737763-supplementary.pdf]

bioengineering

MDPI

Enhancing Trauma Care: Machine Learning-Based Photoplethysmography Analysis for Estimating Blood Volume During Hemorrhage and Resuscitation

Jose M. Gonzalez †, Lawrence Holland †, Sofia I. Hernandez Torres, John G. Arrington, Tina M. Rodgers and Eric J. Snider *

U.S. Army Institute of Surgical Research, JBSA Fort Sam Houston, San Antonio, TX 78234, USA
* Correspondence: eric.j.snider3.civ@health.mil; Tel.: +1-210-539-8721
† These authors contributed equally to this work.

Supplementary Information

Table S1. Summary Results for the Effects of Sampling Window on Model performance. Results for 4 different window sizes – 5-, 10-, 30-, and 60-seconds – and four model architectures are shown – ENET, RF, XGB, and SVR. Average results across all subjects are shown for MAE, MSE, and R^2. Bold R^2 values represent the top performing configuration for each model while the green and red shading indicate the models that were selected or removed from the next optimization steps, respectively.

Sampling Window	5-seconds			10-seconds		
Model	MAE	MSE	R^2	MAE	MSE	R^2
XGB	0.0909 ± 0.059	0.0171 ± 0.026	**0.8392 ± 0.138**	0.0912 ± 0.062	0.0185 ± 0.026	0.825 ± 0.154
RF	0.0845 ± 0.063	0.0173 ± 0.027	**0.846 ± 0.186**	0.0794 ± 0.063	0.0172 ± 0.029	0.844 ± 0.147
ENET	0.128 ± 0.109	0.0344 ± 0.069	0.731 ± 0.113	0.130 ± 0.109	0.0361 ± 0.069	0.719 ± 0.142
SVR	0.0908 ± 0.052	0.0170 ± 0.024	**0.783 ± 0.173**	0.0930 ± 0.050	0.0166 ± 0.019	0.772 ± 0.187
Average	0.0986 ± 0.026	0.0215 ± 0.022	0.799 ± 0.033	0.0984 ± 0.026	0.0221 ± 0.022	0.789 ± 0.020
Sampling Window	30-seconds			60-seconds		
Model	MAE	MSE	R^2	MAE	MSE	R^2
XGB	0.0891 ± 0.062	0.0186 ± 0.029	0.816 ± 0.190	0.0830 ± 0.057	0.0156 ± 0.024	0.839 ± 0.173
RF	0.0831 ± 0.068	0.0185 ± 0.035	0.799 ± 0.236	0.0798 ± 0.064	0.0167 ± 0.032	0.834 ± 0.194
ENET	0.137 ± 0.151	0.0491 ± 0.131	0.737 ± 0.161	0.117 ± 0.083	0.0274 ± 0.039	**0.763 ± 0.142**
SVR	0.0910 ± 0.051	0.0186 ± 0.022	0.738 ± 0.214	0.0967 ± 0.053	0.0183 ± 0.021	0.722 ± 0.231
Average	0.100 ± 0.046	0.0262 ± 0.051	0.773 ± 0.032	0.0940 ± 0.014	0.0195 ± 0.008	0.789 ± 0.038

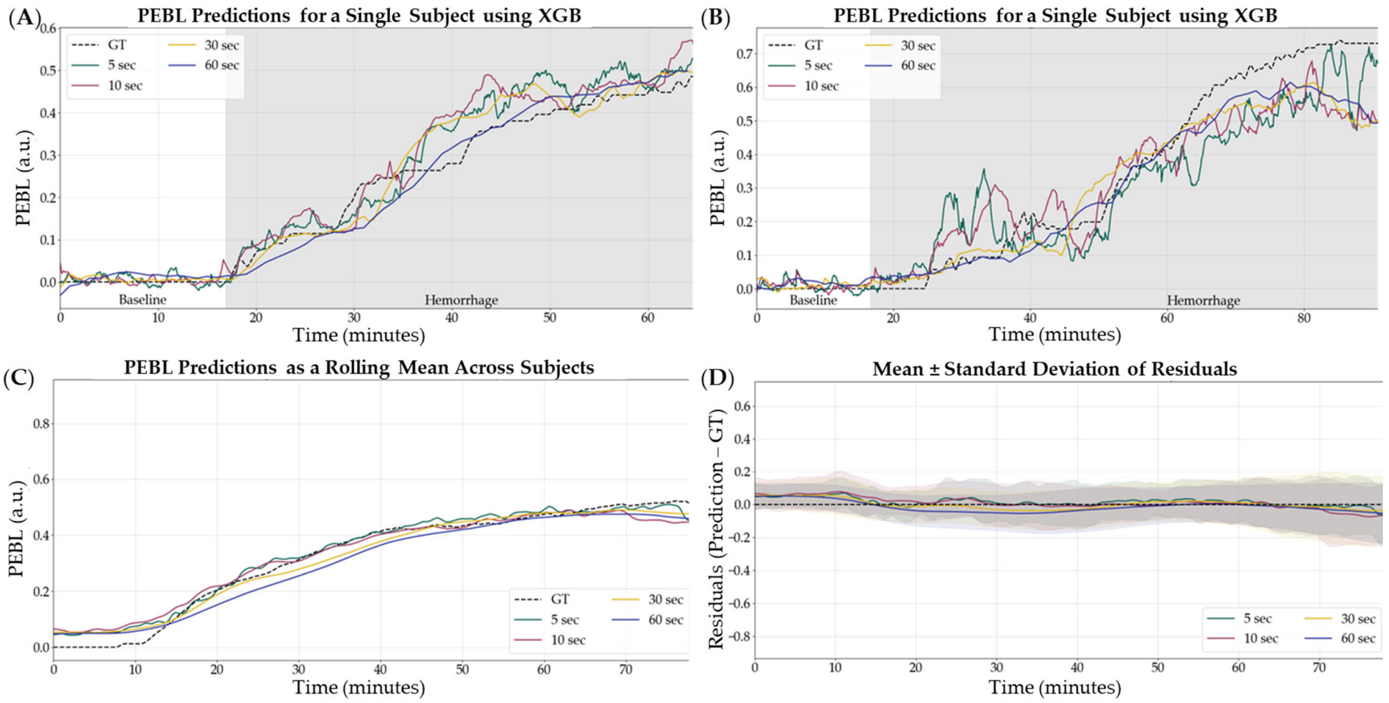

Figure S1. Effect of sample window size on Extreme Gradient Boosting model performance. Four different sampling window sizes were evaluated – 5-, 10-, 30-, and 60-second – compared against calculated GT estimates. (A,B) Representative single subject results for PEBL vs. time during the baseline and hemorrhage scenarios. (C) Average results for XGB for all swine subjects for each sample window size. (D) Average residuals across all subjects for XGB models, with shaded regions denoting standard deviation.

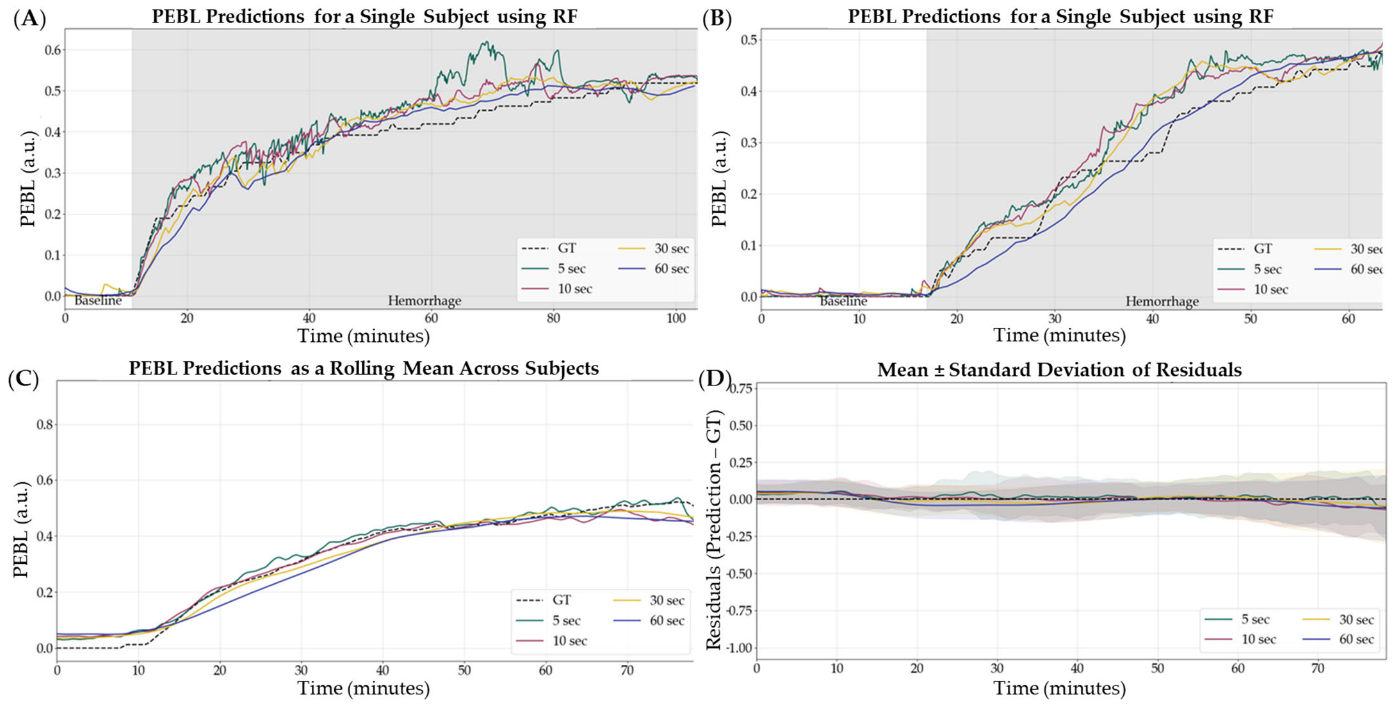

Figure S2. Effect of sample window size on Random Forest model performance. Four different sampling window sizes were evaluated – 5-, 10-, 30-, and 60-second – compared against calculated GT estimates. (A,B) Representative single subject results for PEBL vs. time during the baseline and hemorrhage scenarios. (C) Average results for RF for all swine subjects for each sample window size. (D) Average residuals across all subjects for RF models, with shaded regions denoting standard deviation.

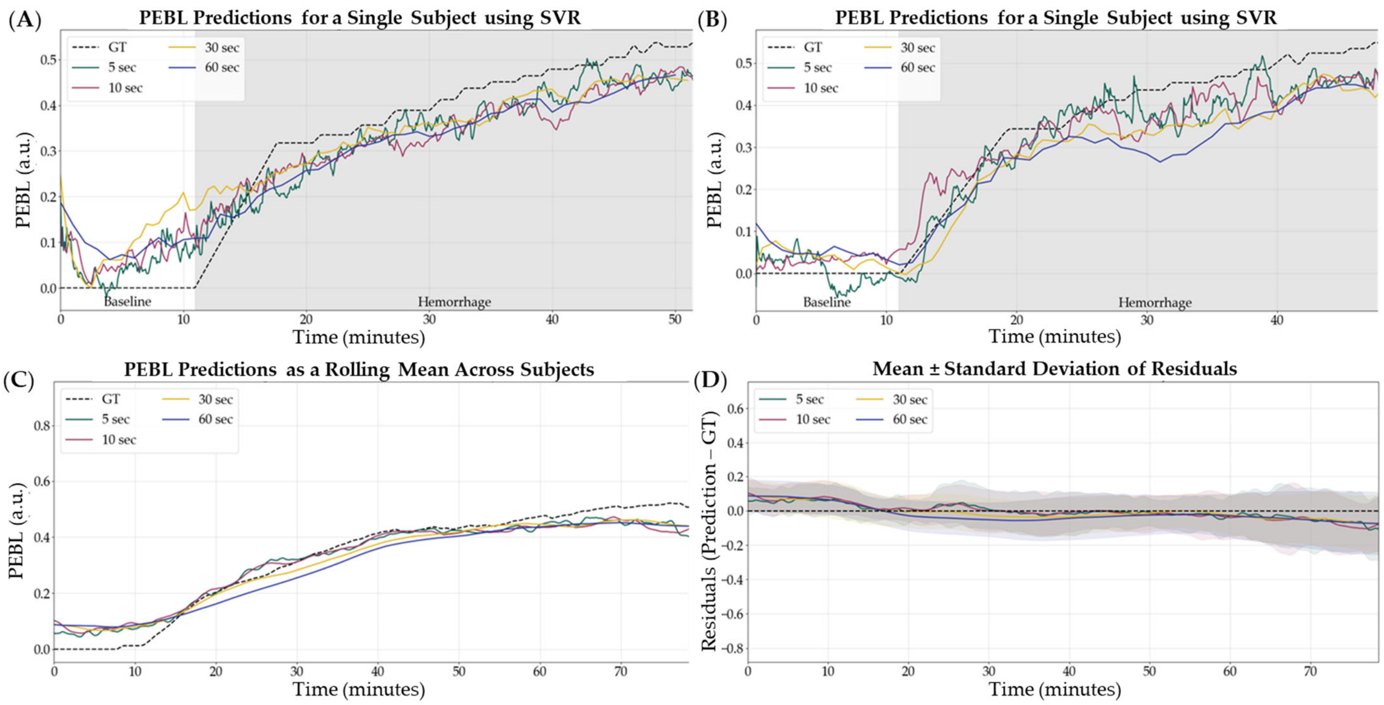

Figure S3. Effect of sample window size on Support Vector Regression model performance. Four different sampling window sizes were evaluated – 5-, 10-, 30-, and 60-second – compared against calculated GT estimates. (A,B) Representative single subject results for PEBL vs. time during the baseline and hemorrhage scenarios. (C) Average results for SVR for all swine subjects for each sample window size. (D) Average residuals across all subjects for SVR models, with shaded regions denoting standard deviation.

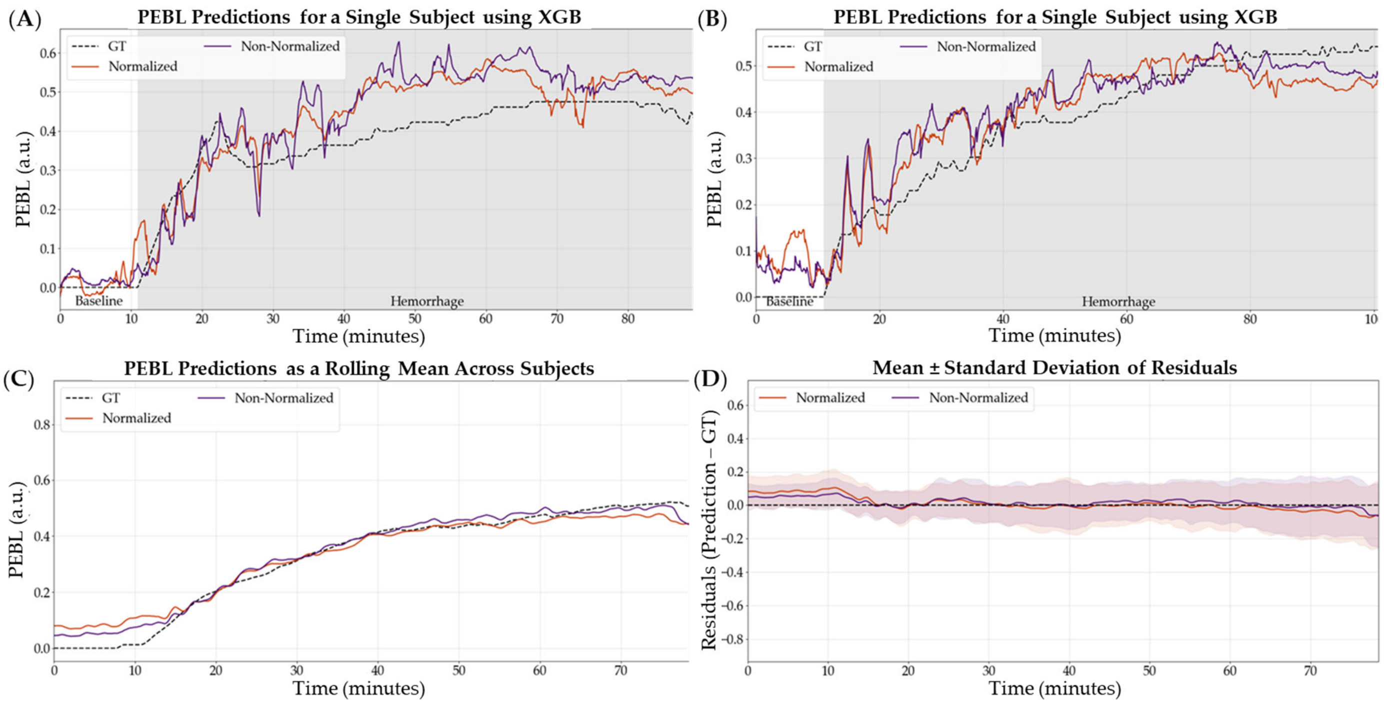

Figure S4. Effect of normalization vs non-normalization on the Extreme Gradient Boosting model performance using a 5 second feature window. (A,B) Representative single subject results for PEBL vs. time during baseline and hemorrhage

scenarios. (C) Average results for XGB for all swine subjects for each normalized and non-normalized window segment length. (D) Average residuals across all subjects for XGB models, with shaded regions denoting standard deviation.

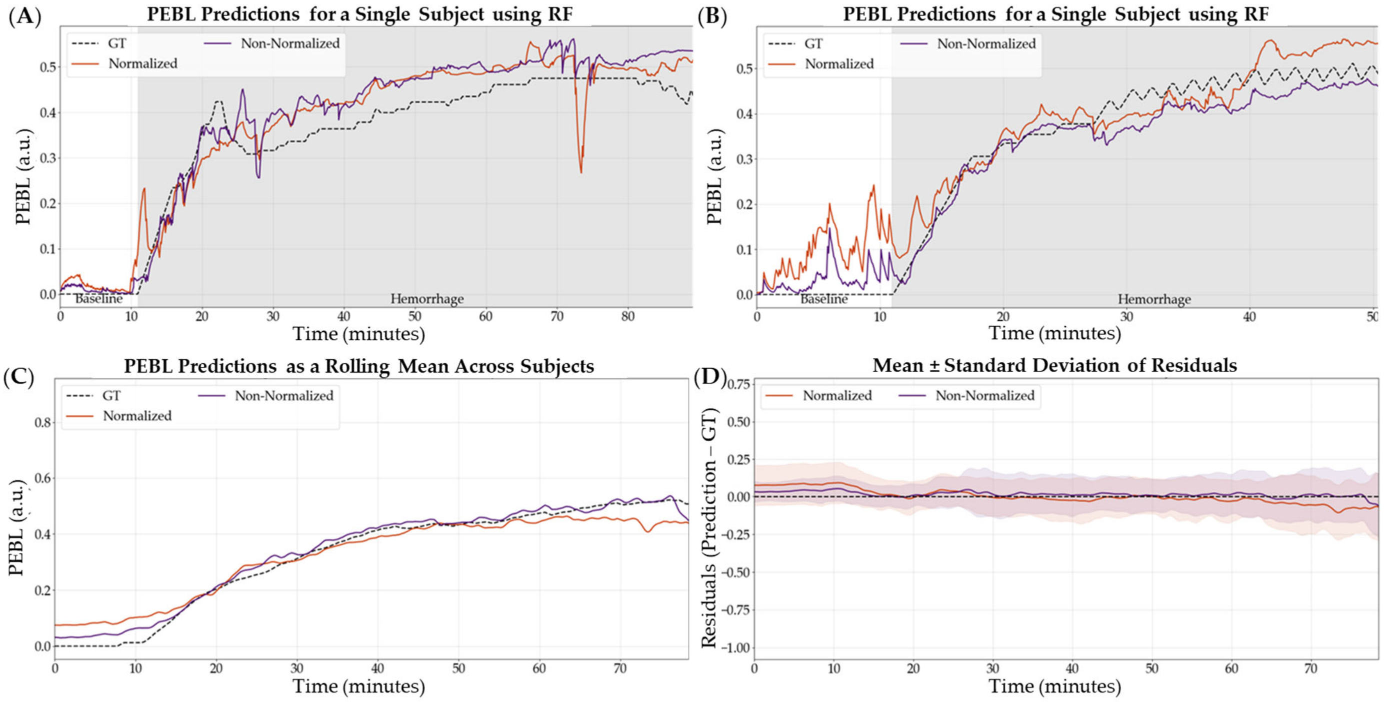

Figure S5. Effect of normalization vs non-normalization on the Random Forest model performance using a 5 second feature window. (A,B) Representative single subject results for PEBL vs. time during baseline and hemorrhage scenarios. (C) Average results for RF for all swine subjects for each normalized and non-normalized window segment length. (D) Average residuals across all subjects for RF models, with shaded regions denoting standard deviation.

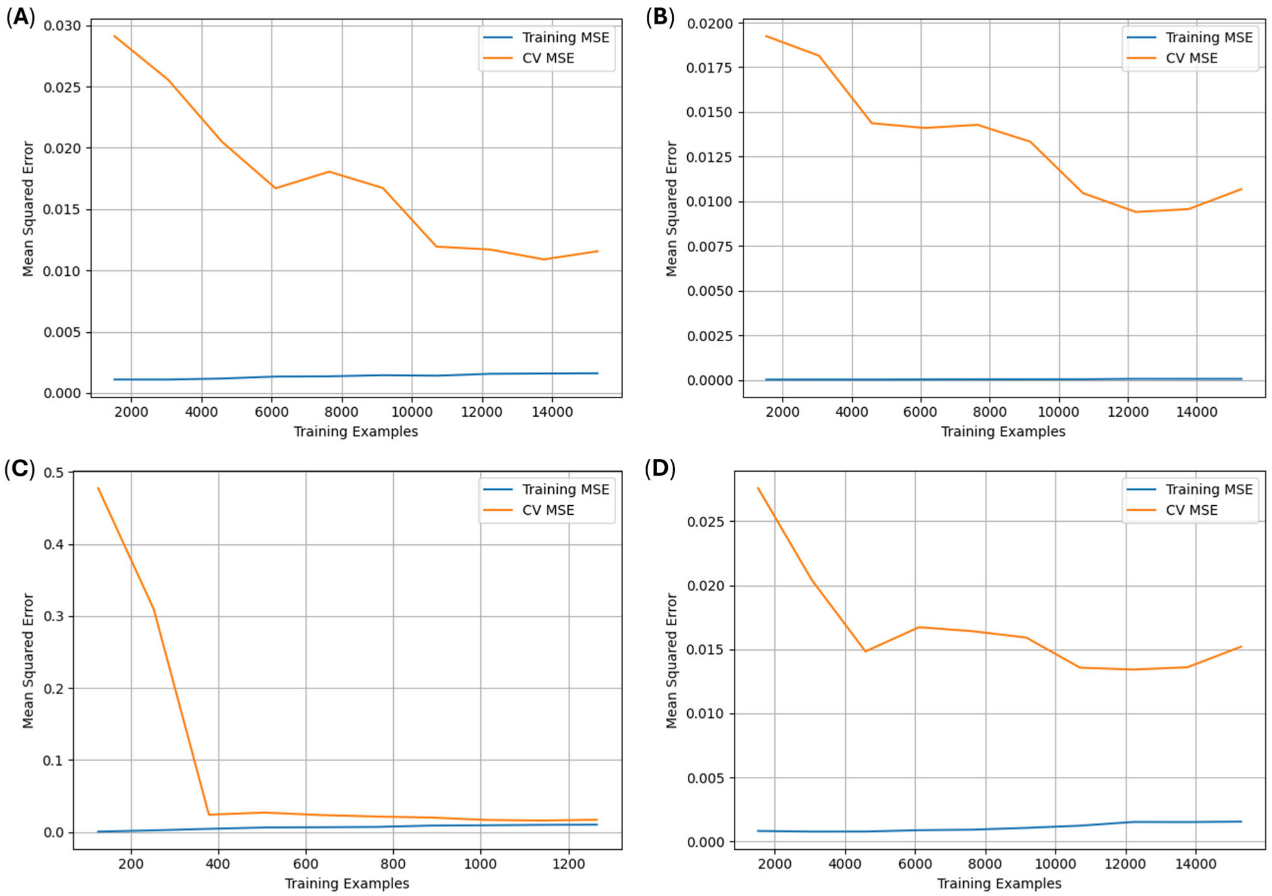

Figure S6. Representative Training Curves for (A) XGB, (B) RF, (C) ENET, and (D) SVR models.
